# High-Throughput Screening and Rapid Inhibitor Triage Using an Infectious Chimeric Hepatitis C Virus

**DOI:** 10.1371/journal.pone.0042609

**Published:** 2012-08-06

**Authors:** Michael J. Wichroski, Jie Fang, Betsy J. Eggers, Ronald E. Rose, Charles E. Mazzucco, Kevin A. Pokornowski, Carl J. Baldick, Monique N. Anthony, Craig J. Dowling, Lauren E. Barber, John E. Leet, Brett R. Beno, Samuel W. Gerritz, Michele L. Agler, Mark I. Cockett, Daniel J. Tenney

**Affiliations:** Bristol-Myers Squibb Research and Development, Wallingford, Connecticut, United States of America; Rosalind Franklin University of Medicine and Science, United States of America

## Abstract

The recent development of a Hepatitis C virus (HCV) infectious virus cell culture model system has facilitated the development of whole-virus screening assays which can be used to interrogate the entire virus life cycle. Here, we describe the development of an HCV growth assay capable of identifying inhibitors against all stages of the virus life cycle with assay throughput suitable for rapid screening of large-scale chemical libraries. Novel features include, 1) the use of an efficiently-spreading, full-length, intergenotypic chimeric reporter virus with genotype 1 structural proteins, 2) a homogenous assay format compatible with miniaturization and automated liquid-handling, and 3) flexible assay end-points using either chemiluminescence (high-throughput screening) or Cellomics ArrayScan™ technology (high-content screening). The assay was validated using known HCV antivirals and through a large-scale, high-throughput screening campaign that identified novel and selective entry, replication and late-stage inhibitors. Selection and characterization of resistant viruses provided information regarding inhibitor target and mechanism. Leveraging results from this robust whole-virus assay represents a critical first step towards identifying inhibitors of novel targets to broaden the spectrum of antivirals for the treatment of HCV.

## Introduction

An estimated 170 million people worldwide are infected with the hepatitis C virus (HCV) [1], [2]. Chronic HCV infection can lead to cirrhosis and hepatocellular carcinoma and is a major cause of liver failure leading to transplantation [3], [4]. Recently, two direct-acting antivirals (DAA), which inhibit the HCV protease, have been approved for therapy, in combination with the previous standard of care, pegylated interferons and ribavirin [5]. These combinations containing DAAs have increased the sustained virological response (SVR) for patients infected with genotype 1 HCV [6]. These are still interferon-containing regimens, the parenteral administration of which can result in severe side effects. Emerging clinical data supports the theory that successful interferon-sparing therapies containing combinations of DAAs can overcome the rapid emergence of resistance and lead to sustained virological response (SVR) [7]. Continued screening and discovery efforts will focus on identifying and combining inhibitors with distinct targets and resistance profiles in order to avoid the emergence of on-treatment resistance as well as to treat patients that developed resistance to prior therapies.

Historically, target selection for HCV drug discovery efforts has been dictated by the availability of surrogate models that recapitulate various aspects of the virus life cycle. For example, genome replication targets (NS3, NS4A, NS4B, NS5A and NS5B) originally became accessible through the development of *in vitro* enzyme and subgenomic replicon assays. As a result, NS3, NS5A and NS5B therapies now dominate the HCV clinical landscape. However, nearly one third of the HCV genome encodes functions not accessible in the replicon system, namely packaging of replicated genomes and assembly into virions, as well as their release, spread to, and entry into new cells. Many of these activities are encoded within structural proteins Core, E1, and E2 acting either alone or in concert with non-structural proteins. Inhibitors directed towards these targets could provide valuable components of an HCV antiviral therapy. For example, potent HCV entry inhibitors, discovered using pseudovirus systems, can block both the entry and spread of infectious virus in cell culture [8], [9]. Additionally, HCV Core dimerization inhibitors [10], [11], [12], identified using an *in vitro* biochemical assay [13], can block the production of infectious HCV in cell culture. Despite these significant advances, numerous other functions mediated by structural proteins (and non-structural proteins) such as nucleocapsid uncoating and the majority of events surrounding virus assembly and release remain largely unchallenged.

Recently, several advances in the HCV cell culture system have been achieved. The growth properties of the JFH1 virus have been improved significantly through adaptive mutations [14], [15], [16] and the generation of an intragenotypic (2a/2a) chimera, referred to as the Jc1 virus [17], [18]. The Jc1 virus produces high titers and can spread rapidly through human hepatocarcinoma cell lines and has been used to successfully develop virus growth assays and screens [19], [20], [21], [22]. Next, chimeric viruses with genotype 1 structural protein coding sequences fused to JFH1 non-structural regions were produced [16], [18], followed by chimeras with structural proteins from each HCV genotype [14], [18], [23], [24], [25], [26], [27]. Genotype 1 infections are the most common worldwide, and are most recalcitrant to interferon-containing therapy. Therefore, inhibitor activity against genotype 1 is a prerequisite for any novel DAA to enter clinical development. Novel HCV DAAs often exhibit selectivity for the genotype or subtype of the virus used for screening necessitating significant medicinal chemistry efforts to achieve broader genotype coverage. In addition, high-throughput screening (HTS) is often facilitated using viruses containing reporter gene proteins, such as luciferase. However, the intergenotypic HCV viruses, and those with reporter genes, often replicate to lower titers and with slower kinetics than those needed for extensive drug discovery. While a full-length genotype 1 clone with robust growth properties has yet to be developed [28], intergenotypic chimeras, where Core-NS2 of JFH1 is replaced with the corresponding region from genotype 1, are a potential source of viruses that can be adapted for comprehensive drug discovery activities. Despite their delayed growth kinetics relative to Jc1 [18], these viruses represent powerful tools for drug discovery since the entire early stage (i.e., virus entry and nucleocapsid uncoating) of the virus life cycle is mediated by genotype 1 proteins while virus assembly is orchestrated by a combination of genotype 1 and 2 proteins.

Here, we report on the use of a genotype 1a/2a chimeric, reporter virus to develop a robust, homogeneous, high-throughput, multi-cycle virus replication assay and demonstrate its capability in HTS of a large-scale, small-molecule compound library. This novel screening approach was validated using a comprehensive array of secondary assays that classified hits according to potency, selectivity, life cycle stage targeted and genotype coverage.

## Results

### Development and validation of a multi-cycle virus growth assay

**Figure 1:**
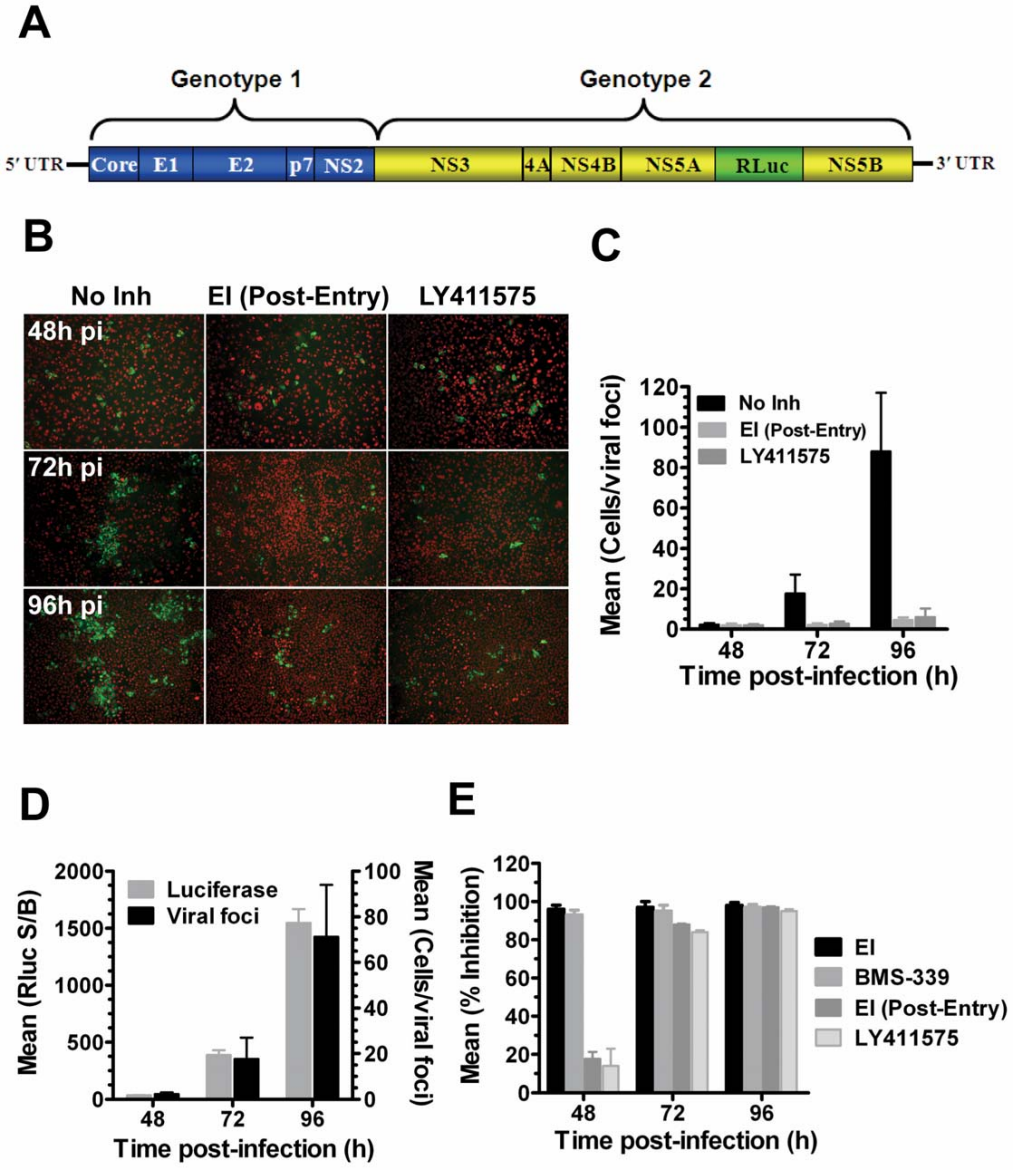
The gt 1a/2a-Rluc virus is capable of multi-cycle virus growth amenable to unbiased inhibitor detection. A. Schematic of the genotype 1a/2a-Rluc virus which harbors Core-NS2 from the genotype 1a H77 isolate fused to NS3-NS5B of the genotype 2a JFH1 isolate with an Rluc reporter gene cloned between NS5A and NS5B. B–E. Huh-7.5 cells were infected with gt 1a/2a-Rluc (MOI = 0.05) and processed for HCV Core immunofluorescence or *Renilla* Luciferase expression 48, 72 and 96 h post-infection (pi). Virus spread was inhibited using an HCV entry inhibitor (EI; 1 µM) added 16 h post-entry [EI (Post-Entry)] or with an SPP inhibitor (LY411575; 0.5 µM). Virus infectivity and spread were assessed directly using immunofluorescence microscopy of HCV Core (green) and Huh-7.5 nuclei (red) to calculate the number of infected cells per viral foci (B, C, & D) or indirectly with *Renilla* Luciferase (D & E). Results are expressed as the mean and standard deviation of at least two independent assays.

An intergenotypic (1a/2a) reporter (*Renilla* luciferase; Rluc) virus, engineered and adapted for high-titer replication and spread [8] was used to develop a multi-cycle virus growth assay. The gt 1a/2a-Rluc virus harbors genotype 1a (H77) Core-NS2, genotype 2a (JFH1) NS3-NS5B and a stable Rluc cassette inserted intergenically between NS5A and NS5B (Fig. 1A) and typically yields titers of 1–5×10^5^ focus forming units (ffu)/ml (data not shown). As a first step in developing a screening assay capable of detecting inhibitors at all stages of the virus life cycle, the spreading kinetics of the gt 1a/2a-Rluc virus was characterized in Huh-7.5 cells. To discriminate between enlarging foci due to virus spread and those due to cell division alone, control cultures included inhibitors that block either virus entry or assembly. A virus entry inhibitor capable of blocking both entry and cell-cell spread (EI [8]) was added to cultures 16 h following the initial infection (EI added post-entry; EI^PE^) to simulate a late-stage inhibitor through its ability to block virus spread. A signal peptide peptidase (SPP) inhibitor, LY411575, which blocks the SPP-mediated processing/maturation of Core, was used to block the release of infectious virus [29], [30], [31], [32], [33], [34], [35], [36], [37], [38], [39], [40]. The results showed that HCV Core-positive virus foci were detectable within 48 h post-infection (pi) and their size (2.2±0.8 mean cells/foci) was indistinguishable from cultures in which spread was inhibited with EI^PE^ (1.9±0.8) or LY411575 (2.0±0.6; Figs. 1B & C), suggesting that virus spread was not detectable at this time point. Expansion of virus foci was typically observed within 72 h (17.6±9.4) and large foci (88.0±29) were observed at 96 h pi (Figs. 1B & C). The expansion of virus foci was blocked by both EI^PE^ and LY411575 with >20 fold inhibition observed at the 96 h time point (Figs. 1B & C). Importantly, the expansion of virus foci correlated with an increase in *Renilla* luciferase expression (Fig. 1D). Consistent with the results above, EI^PE^ and LY411575 exhibited only modest inhibition (<20%) of luciferase expression at 48 h pi but achieved >80% at 72 h and >90% inhibition at 96 pi (Fig. 1E). As expected, addition of the entry inhibitor or an NS3 protease inhibitor (BMS-339) at the time of infection inhibited luciferase expression at all time points (Fig. 1E). Taken together, these results suggested that a 96 h incubation period was necessary and sufficient for unbiased identification of inhibitors of all phases of viral replication using the gt 1a/2a-Rluc virus. Our goal was to develop an assay where >90% of the Luciferase signal was due to virus spread so as to avoid bias towards early or genome replication inhibitors. While it has been reported that earlier time points are optimal for spreading of fully genotype 2a viruses, Figure 1 shows that a 96 h incubation period was required to achieve this goal. These results further demonstrated that single and multi-cycle virus replication could be delineated by monitoring luciferase expression at either the 48 or 96 h pi time points, respectively.

### Transition to a homogenous, 384 well format assay

**Figure 2:**
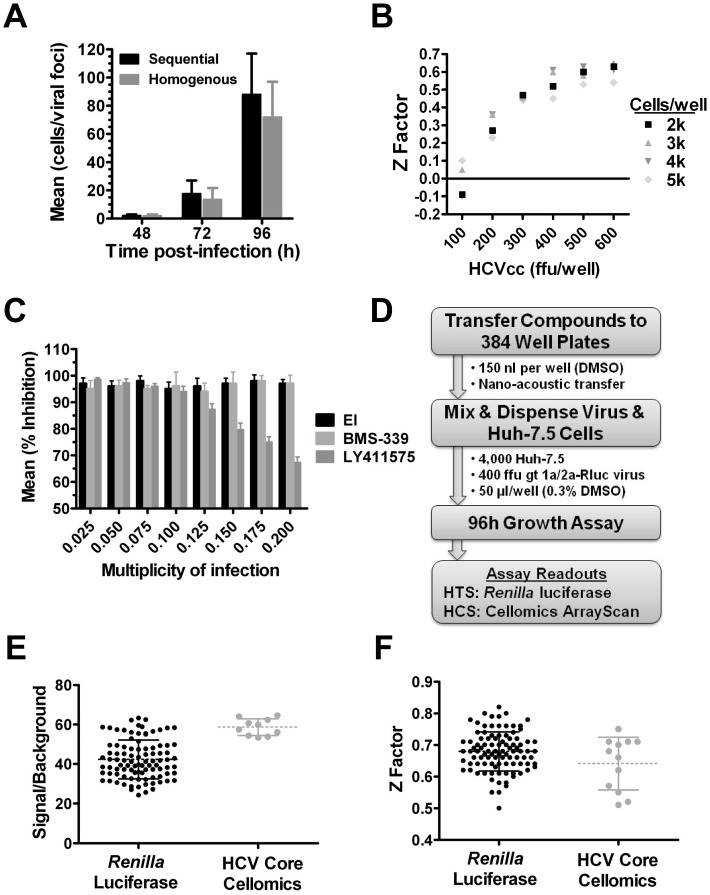
Optimization of a high-throughput 384 well virus replication assay. A. Comparison of virus replication over time (cells/viral foci) following infection using a standard protocol where virus was added to adherent Huh-7.5 cells (pre-plated 24 h prior to infection) or a homogenous protocol where virus and trypsinized cells were co-dispensed into a well. B. Co-titration of genotype 1a/2a-Rluc virus and Huh-7.5 cells to determine Z factor values in a 96 h assay. C. Effect of MOI on % inhibition obtained with entry (EI), genome replication (BMS-339) and virus assembly (LY411575) inhibitors in a 96 h assay. D. HTS assay strategy. E & F. HTS assay quality control. Plate-to-plate variability in signal/backgroundr (E) and Z factor (F) was determined for the *Renilla* luciferase and HCV Core Cellomics ArrayScan readouts from 50 and 12 plates validation runs, respectively. Results are expressed as mean and standard deviation.

Rapid screening of large-scale compound libraries requires homogenous, miniaturized platforms that can be automated. A first step towards assay simplification was to determine if virus and trypsinized cells could be mixed in suspension to initiate infection, and dispensed onto plates containing screen compounds, instead of adding virus to adherent cells plated 24 hours prior. It was observed that the effective titer of a virus stock (ffu per well) was typically reduced approximately 1.5-fold in the one-step procedure relative to a multi-step infection protocol (data not shown) and the expansion of viral foci (virus per foci) was only modestly (<2 fold) delayed (Fig. 2A). Importantly, the homogenous protocol had no impact on the potency of control inhibitors (data not shown).

A low MOI is necessary to enable the multiple rounds of replication required to interrogate all replication stages. However, the MOI needs to be high enough to achieve a robust signal/background with minimal well-to-well variability. This signal/variability relationship is referred to as the Z factor [41] and is generally preferred to be ≥0.5 to enable statistically reliable identification of inhibitors during HTS. First, the optimal ratio of gt 1a/2a-Rluc virus to Huh-7.5 cells in a 384 well plate format was determined. While cell numbers influenced Z factor somewhat, the amount of virus used had a more dramatic effect, with ≥400 ffu of virus/well and 4,000 Huh7.5 cells providing an acceptable Z factor (Fig. 2B). With 400 ffu of virus/well and 4,000 Huh7.5 cells (MOI = 0.1), entry (EI), replication (BMS-339) and late-stage (LY411575) inhibitors all achieved >90% inhibition in a 96 h assay (Fig. 2C). As expected, increasing the input of virus (MOI>0.1) reduced the percent inhibition for the late-stage inhibitor (Fig. 2C) confirming that when using 4,000 Huh-7.5 cells/well an MOI of 0.1 or lower was critical to ensuring detection of late-stage inhibitors. Additional experiments were performed to enable HTS, including studies that showed that the gt 1a/2a-Rluc virus (and other HCVcc viruses) was amenable to large-scale preparation and storage at −80°C and that Huh-7.5 cells could be cultured on a preparative-scale using robotics without loss of HCVcc permissiveness (data not shown).

Figure 2D outlines the strategy used for HTS. Virus and freshly harvested Huh-7.5 cells were pre-mixed at a MOI of 0.1 and 50 µl/well was dispensed into 384 well plates harboring 150 nl of compound/well in DMSO (0.3% final). It was determined empirically that virus infectivity was negatively affected by DMSO concentrations exceeding 0.3% (data not shown). Assay plates were incubated for 96 h and luciferase expression was measured using EnduRen™, a live-cell *Renilla* luciferase substrate. An alternative Cellomics ArrayScan high-content screening (HCS) readout was also developed for the purpose of confirming HCV activity for screen hits identified from the luciferase HTS (see Methods). This alternative assay format involved immuno-staining of HCV core protein and Hoechst labeling of nuclei to determine the percent of infected cells relative to the total number of cells in the culture (HCV Core Cellomics Assay). Test runs for *Renilla* luciferase (100 plates) and HCV Core Cellomics (12 plates) assays yielded mean signal/background values of 41.2±9.81 and 58.6±4.21 (Fig. 2E), respectively, as well as Z factor values of 0.67±0.06 and 0.64±0.08, respectively (Fig. 2F). Importantly, the EC_50_ values for established HCV inhibitors including entry (EI), NS3 protease (BMS-339), NS5A (BMS-052), NS5B (IDX184), Interferon α, and SPP (LY411575) were similar between the HTS and HCS readouts (Table 1). Together, these results showed that both assays were robust, reproducible and suitable for large-scale screening. The *Renilla* luciferase readout was used to screen a compound library consisting of 1,175,504 compounds. Nineteen runs (≥150 plates per run) were performed and the average Z factor for the HTS campaign was 0.58±0.02 (data not shown). Throughput for this assay was approximately 150,000–200,000 compounds per week.

**Table 1:** Assay Performance With Known HCV Inhibitors.

Inhibitor	Lifecycle stage/target	Luciferase^a^ (EC_50_, nM)	Cellomics^a^ (EC_50_, nM)
EI	Entry/E2	33±6.3	31±9.5
BMS-339	Replication/NS3	256±25	270±22
BMS-052	Replication/NS5A	0.015±0.001	0.016±0.001
IDX184	Replication/NS5B	2,901±237	2,644±256
IFNα	Replication/host response	0.20±0.04^b^	0.17±0.08^b^
LY411575	Assembly/SPP^c^	10±3.3	11±4.1

a Average EC_50_ (nM) ± standard deviation of at least three experiments.

b International units per ml.

c SPP, Signal peptide peptidase.

### HTS Identified Inhibitors of all Stages of the Virus Life Cycle

**Figure 3:**
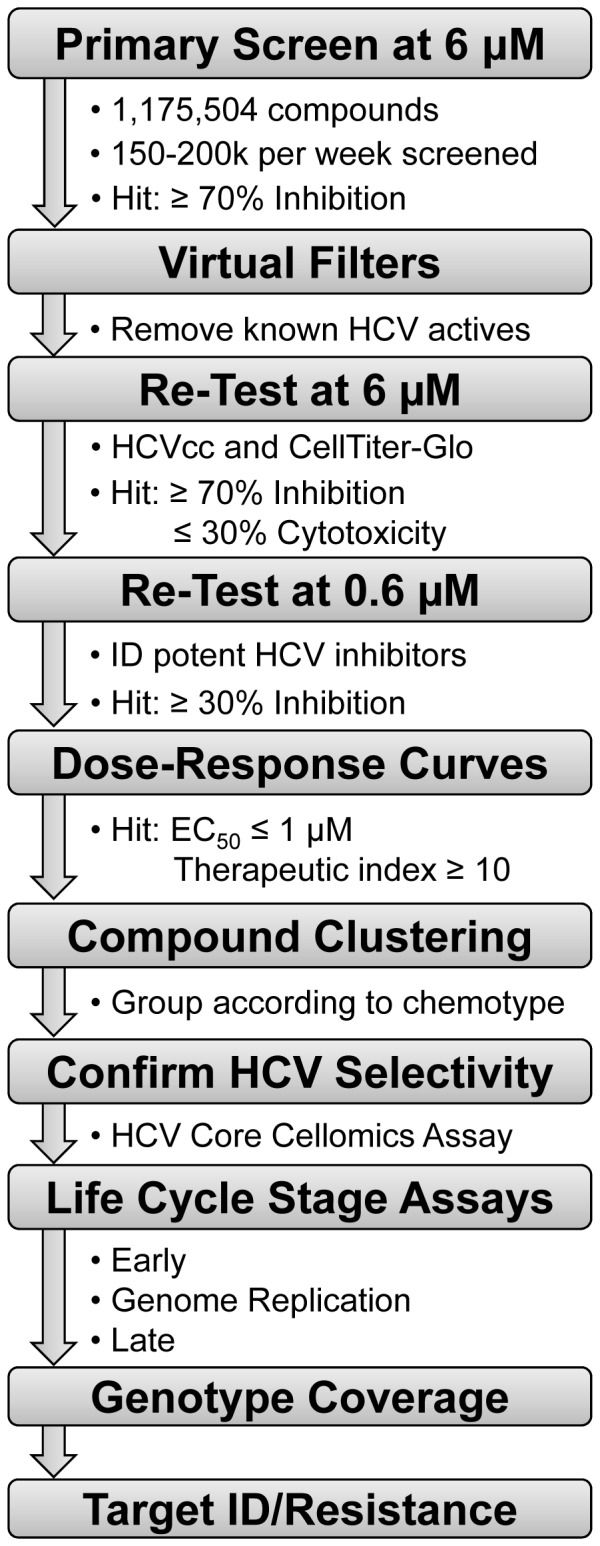
Outline of primary screen, re-test and hit triage strategy. Potent and selective hits identified from a primary screen were subjected to a myriad of life cycle stage assays, genotype coverage and resistance studies to group hits according to target.

**Figure 4:**
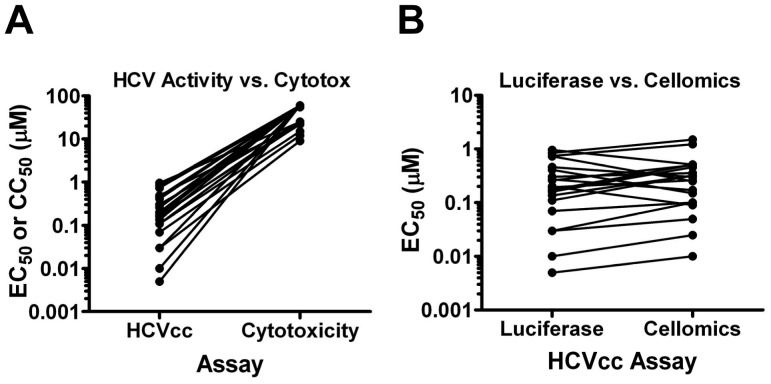
Confirmation of anti-HCV activity for top 17 hits. A. Potency (EC_50_) against the gt 1a/2a-Rluc virus (EC_50_) in a 96 h multi-cycle assay and corresponding Huh-7.5 cell cytotoxicity (CC_50_) for each of the top 17 screen hits. B. Confirmation of anti-HCV activity through comparison of potency using *Renilla* Luciferase or HCV Core Cellomics ArrayScan readouts.

Figure 3 outlines the strategy to triage potent and selective leads and rapidly categorize screen hits. This included removing known HCV actives, and selections based on potency and lack of cytotoxicity, as well as confirming activity. These steps identified actives for which further assay results were less likely to be confused with subtle cytotoxicity. Then secondary assays such as compound chemotype clustering and assays that distinguish compounds based on the mechanism of activity were used. The results of the screen are presented in Table 2. Primary screening (1,175,504 compounds) and re-tests identified 9,025 compounds (0.77% hit rate) with ≥70% inhibition of HCV at 6 µM and ≤30% cytotoxicity as measured by CellTiter-Glo (Table 2). To expeditiously identify the more potent leads (e.g., EC_50_≤1 µM), hits were re-tested at 0.6 µM (0.1× initial screening concentration) and 632 compounds were selected based on ≥30% inhibition. Dose-response curves were used to determine EC_50_, as well as the 50% cytotoxicity concentration (CC_50_). There were 386 compounds with an EC_50_≤1 µM and 307 compounds with a therapeutic index (TI) ≥10 (CC_50_/EC_50_). To ensure none of the screen hits directly affected *Renilla* luciferase, the compounds were tested against Vesicular stomatitis virus (VSV) pseudo-typed retroviral particles harboring either a firefly or *Renilla* luciferase reporter. Seventy five compounds were eliminated and the remaining 232 screen hits were clustered based on similarity using atom pair descriptors and in-house software. Seventeen compounds, each representing a unique chemical chemotype, were advanced for further analysis. Therapeutic indices for these hits ranged from 22 to >6,000 (Fig. 4A) and each demonstrated similar potency using either the *Renilla* luciferase or HCV Core Cellomics readouts confirming their specificity for HCV (Fig. 4B).

**Table 2:** Primary Screen Results.

Screening Tier	Criteria	Pass	Fail
Primary Screen & Re-Tests^a^	≥70% inhibition/≤30% cytotoxicity at 6 µM compound. Removed known HCV actives.	9,025	1,166,479
Potency Filter	≥30% inhibition at 0.6 µM compound.	632	8,865
Dose-Response Curves	EC_50_≤1 µM^b^	386	246
Therapeutic Index ≥10^c^	CC_50_/EC_50_ ≥10	307	79
*Renilla* luciferase counterscreen	No activity against *Renilla* luciferase VSV pseudo-typed reporter retrovirus.	232	75
Chemical analysis	Compounds grouped by similarity. Representative for each class advanced.	17	-
Hit Confirmation	Similar EC_50_ using *Renilla* luciferase and Cellomics assay read-outs.	17	0

a Single-point (6 µM); Re-tests in triplicate.

b Mean EC_50_ achieved from 10-point dose-response curve; in triplicate.

c CellTiter-Glo cytotoxicity assay.

### Inhibitor Target Characterization

**Figure 5:**
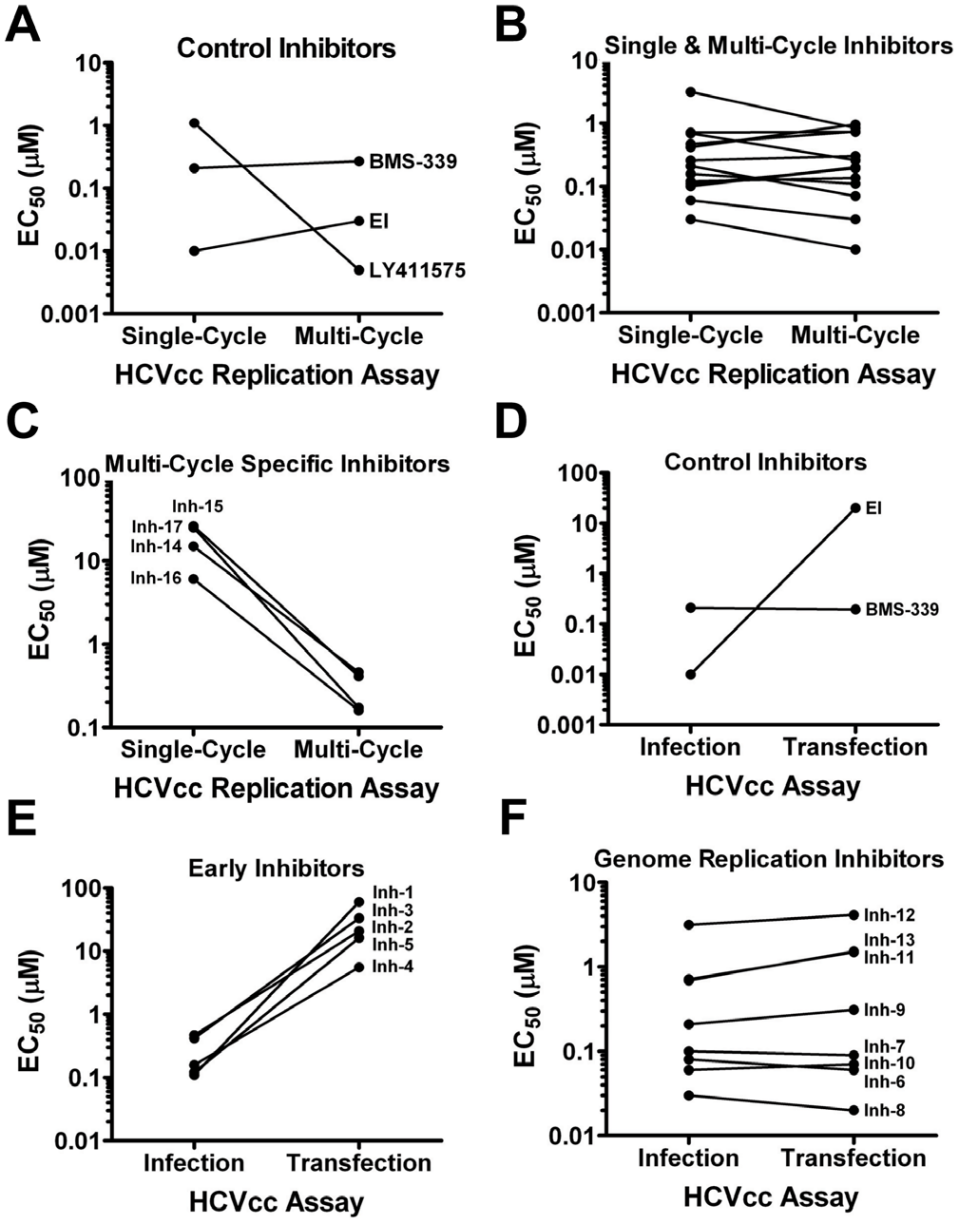
Life cycle stage triage. A. Performance (EC_50_) of control entry (EI), genome replication (BMS-339) and late-stage (LY411575) inhibitors in single- vs. multi-cycle replication assay formats. B. Screen hits (Inhs 1–13) that demonstrated similar potency in single- and multi-cycle replication formats (single- & multi-cycle inhibitors). C. Screen hits (Inhs 14–17) that demonstrated greater potency in multi-cycle replication assay format (multi-cycle specific inhibitors). D. Performance of control entry (EI) and genome replication (BMS-339) inhibitors following infection with gt 1a/2a-Rluc virus or direct transfection of the corresponding virus genomic RNA. E. Screen hits (Inhs 1–5) that demonstrated reduced potency following direct transfection of virus genomic RNA (early inhibitors). F. Screen hits (Inhs 6–13) that demonstrated similar potency in infection and transfection assays (genome replication inhibitors).

Table 3 outlines the assays used to deconvolute screen hits according to virus life cycle stage target and predicts the expected profile for inhibitors targeting early (e.g., entry and nucleocapsid uncoating), genome replication or late (e.g., virus assembly and release) events in the virus life cycle. As a first step in elucidating the stage at which the hits were active, the potencies of the compounds were compared using single and multi-cycle HCVcc infectivity assays. As previously determined (Fig. 1), if the assay was constrained to 48 hours, luciferase activity largely represented the first replication cycle only. In contrast, >90% of the signal after 96 h pi was attributed to subsequent rounds of replication. As expected, control entry (EI) and genome replication (BMS-339) inhibitors exhibited similar potency at 48 and 96 h pi, while the late-stage inhibitor (LY411575) was significantly more potent (>200 fold) at 96 h (Fig. 5A). Of the 17 hits selected for further analysis, 13 showed similar potencies (<3-fold difference) at both 48 and 96 h pi (Fig. 5B) while 4 hits (Inhs 14, 15, 16 & 17) showed a marked increase in potency (32–145 fold) at 96 h (Fig. 5C). These results suggested those 13 inhibitors with similar potencies at 48 and 96 h act coincident with early or genome replication inhibition, while the 4 with increased potency at 96 h with late-stage inhibition.

**Table 3:** Expected outcome for life cycle stage assays.

	HCV Life Cycle Stage			
HCV Assays	Entry	Post-Fusion/Pre-Genome Replication	Genome Replication	Late
HCVcc: Single-Cycle Replication	+	+	+	−
HCVcc: Multi-Cycle Replication	+	+	+	+
HCVpp	+	−	−	−
vRNA Transfection	−	−	+	−
HCV Replicon	−	−	+	−
HCVcc: Infectious Virus Release	−	−	−	+

To discriminate between early and genome replication inhibitors, the entry process was bypassed by direct transfection of viral RNA (vRNA). *Renilla* luciferase signals were measured at 48 h pi of HCVcc or post-transfection of the corresponding vRNA. As expected for an entry inhibitor, EI blocked the luciferase signal following HCVcc infection but not transfection of vRNA, while the control genome replication inhibitor (BMS-339) showed similar potency in both assays (Fig. 5D). Of the 13 hits that showed similar potency in the single- and multi-cycle assays, 5 (Inhs 1–5) were less active following transfection of vRNA (Fig. 5E) while 8 (Inhs 6–13) exhibited similar potency in both formats (Fig. 5F). Altogether, this triage strategy segregated the 17 hits into three temporally & mechanistically-distinct virus replication stage categories including early (5), genome replication (8) and late (4) inhibitors.

**Figure 6:**
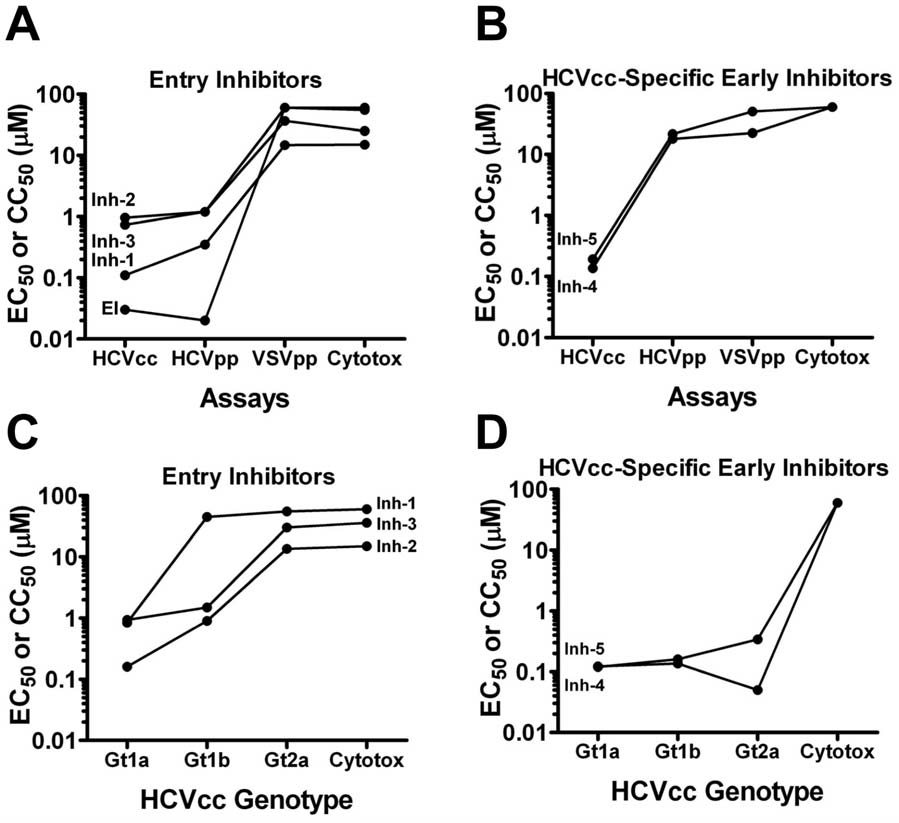
Early/Entry Inhibitors. A. The potencies (EC_50_) of early inhibitors (Inhs 1–5) against gt 1a/2a-Rluc virus (HCVcc) and HCV pseudo-particles (HCVpp) with the corresponding genotype 1 envelope glycoproteins were compared to identify virus entry inhibitors. Selectivity for HCVpp was confirmed using Vesicular stomatitis virus glycoprotein pseudo-particles (VSVpp) and cytotoxicity (CC_50_) using Cell-Titer Glo. A. Screen hits (Inhs 1–3) that demonstrated similar potency against HCVcc and HCVpp and exhibited selectivity relative to VSVpp and cytotoxicity (entry inhibitors). A control entry inhibitor (EI) was included to confirm the predicted outcome for a bonafide entry inhibitor. B. Screen hits (Inhs 4–5) that demonstrated reduced potency against HCVpp (HCVcc-specific early inhibitors). Genotype selectivity was assessed by comparing potency against HCVcc chimeras with genotype 1a, 1b or 2a structural proteins. C. Potency of the entry inhibitors (Inhs 1–3) and (D) HCVcc-specific early inhibitors (Inhs 4 & 5) against genotype 1a, 1b and 2a HCVcc and corresponding cytotoxicity.

To identify entry-specific compounds, the 5 early-stage hits were tested against HCV pseudo-particles (HCVpp) harboring the HCV genotype 1a envelope glycoproteins matching those of the screening virus. Three of the 5 hits exhibited a similar profile as EI with similar potency against both HCVcc and HCVpp viruses (Fig. 6A). HCV selectivity was confirmed by counter-screening for activity against VSVpp and cytotoxicity (Fig. 6A). Together, these findings confirmed that Inh-1, 2 and 3 were HCV entry inhibitors. On the contrary, Inhs 4 and 5 showed no activity against HCVpp (Fig. 6B) suggesting that these hits could target an HCVcc-specific entry event or an early event not recapitulated by the pseudo-particle system (e.g., nucleocapsid uncoating). Next, genotype coverage of the inhibitors was determined using HCVcc chimeras harboring genotype 1a, 1b (432–4 isolate) or 2a (J6 isolate) structural proteins. Of the 3 entry inhibitors, Inh-1 was selective for genotype 1a, while Inhs 2 and 3 exhibited activity against genotype 1a and 1b but not 2a (Fig. 6C). A similar profile was observed using HCVpp harboring genotype 1a,1b or 2a envelopes (data not shown). For the HCVcc-specific inhibitors, both Inh-4 and Inh-5 exhibited similar potency against all 3 genotypes (Fig. 6D).

### Viral Genome Replication Inhibitors

**Figure 7:**
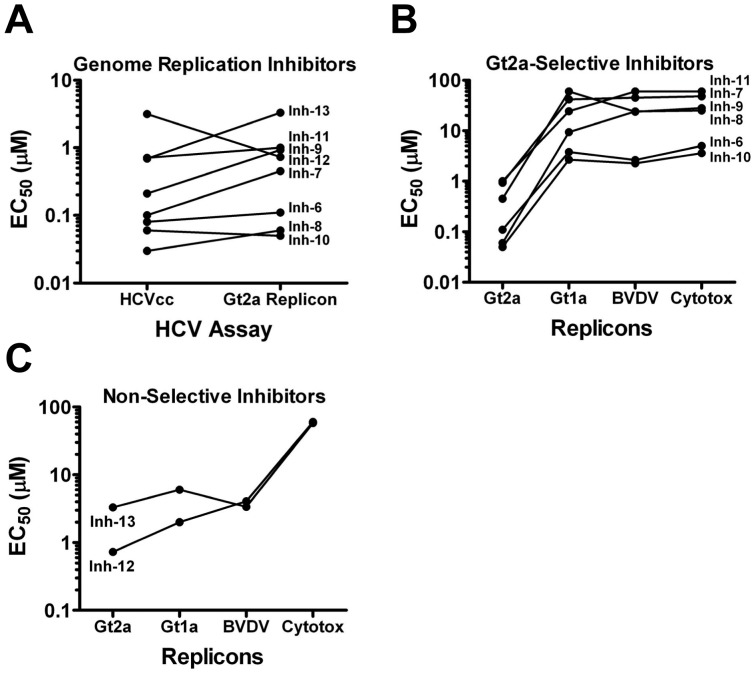
HCV Genome Replication Inhibitors. A. The genome replication inhibitors (Inhs 6–13) were confirmed by comparing potency (EC_50_) against the 1a/2a-Rluc virus and a corresponding JFH1 replicon. B & C. Genotype 2a and HCV selectivity were assessed by comparing inhibitor potency against genotype 2a, genotype 1a and BVDV replicons and compounds were subsequently grouped into genotype 2a-selective (B) and non-selective (C).

HCV and Bovine viral diarrhea virus (BVDV) replicons were used to determine if Inhs 7–13 were HCV-selective genome replication inhibitors. All of the hits exhibited similar potency against HCVcc and a genotype 2a replicon (Fig. 7A), confirming that these compounds block replication of the viral genome. Of the 8 inhibitors, 6 (Inhs 6–11) exhibited reduced potency against both genotype 1a HCV and BVDV replicons which was indistinguishable from cytotoxicity (Fig. 7B) showing that these agents were selective for genotype 2a HCV. On the contrary, Inhs 12 and 13 exhibited similar activity against genotype 2a and 1a HCV as well as BVDV replicons (Fig. 7C) and these activities were separable from cytotoxicity. Taken together, these results demonstrated that these inhibitors were not selective for HCV but rather blocked the replication of both viruses.

### Late Inhibitors

**Figure 8:**
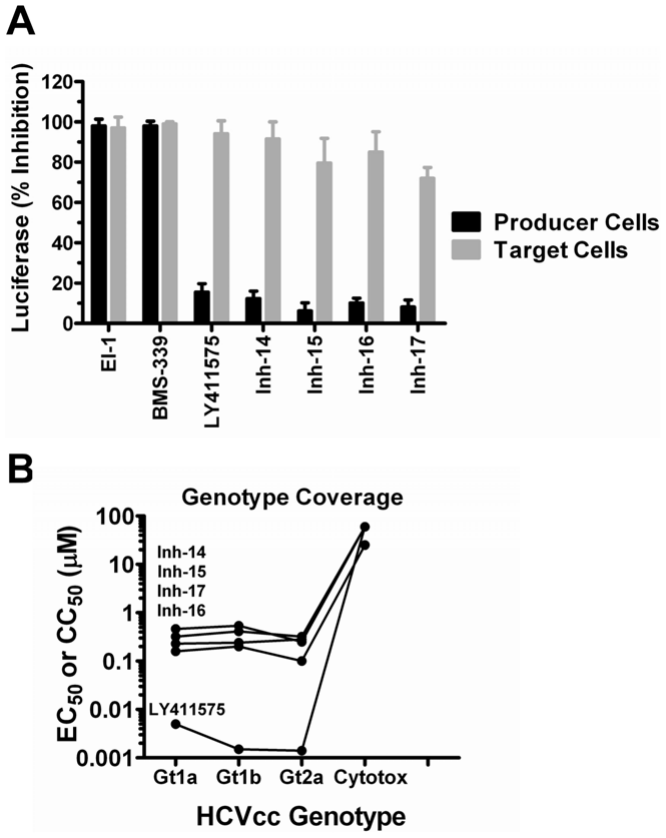
Late Stage Inhibitors. Inhibitors that showed enhanced potency in the multi-cycle virus replication assay (Inhs 14–16) were tested for their ability to block the production of infectious virus. A. Control inhibitors that block virus entry (EI) and genome replication (BMS-339) inhibited *Renilla* luciferase expression in both producer (black bars) and target (gray bars) cells while a control late-stage inhibitor (LY411575) only affected *Renilla* luciferase expression in target cells. Similar to LY411575, Inhs 14–17 exhibited less than 20% inhibition of *Renilla* luciferase expression in producer cells but >75% inhibition in target cells suggesting a block in the production of infectious virus. B. Genotype coverage was assessed by comparing the potency (EC_50_) of Inhs 14–17 and LY411575 against HCVcc with genotype 1a, 1b or 2a structural proteins.

To confirm that Inhs 14–17 targeted a late stage in the virus life cycle, their ability to block production of infectious virus was analyzed. Huh-7.5 cells were infected with HCVcc (MOI = 1) in the presence of 2× EC_90_ of each compound and supernatant from these cells (virus producer cells) was harvested at 48 h pi and transferred to naïve target cells. Luciferase expression was measured in both producer and target cells at 48 h pi. As expected, viral replication was inhibited in producer cells by both the control entry (EI) and replication (BMS-339) inhibitors (Fig. 8A). On the contrary, LY411575 and Inhs 14–17 had no effect on genome replication in infected producer cells but rather inhibited the infection of naïve target cells (Fig. 8A) suggesting that these inhibitors target a late stage of the virus life cycle that affects the production of infectious virus. LY411575, as well as Inhs 14–17, exhibited similar potency against genotype 1a, 1b and 2a HCVcc chimeras (Fig. 8B).

### Resistance selection

Antiviral discovery is aided significantly using cell culture methodology to select for and characterize resistant viral variants. These studies can yield information regarding; the molecular target of inhibition (is it viral or cellular?), if the resistance pattern/profile is unique or overlaps with other antivirals, and if the changes that emerge in the resistant viruses are already present in the population of viruses that are circulating in patients prior to anticipated therapy. We tested the ability of the gt1a/2a chimeric HCVcc virus to be used for the selection of resistant virus variants using a select series of control inhibitors and screen hits and these results along with corresponding genotype coverage are presented in Table 4. EI, an entry inhibitor with genotype 1a and 1b coverage [8] was used as a control for a compound that hits a viral target. As reported previously [8], a V336G substitution in E2 was selected by EI which conferred 45 fold resistance to the drug. In contrast, LY411575, which perturbs HCV core processing by blocking SPP-mediated Core processing, did not select for resistance through ten weeks of culture (Table 4). Of the five screen hits tested, resistance was observed with Inhs 2, 6 and 7 while no resistance was observed for Inhs 4 and 17. Two independent populations were obtained for Inh-2, the genotype 1-selective entry inhibitory (Fig. 6), and whole-genome sequencing revealed one population had an L67F substitution in E1 while the other had a W333L substitution in E2 (Table 4). These substitutions conferred complete resistance by themselves (Table 4) or in combination (data not shown). Two populations exhibiting 30 and 100 fold resistance to Inh-6 were selected and sequencing revealed mutations clustered exclusively within NS5A. A combination of F28L, L31M, and F169L conferred 30 fold resistance while a combination of L31M, S38T, and Q123R conferred 100 fold resistance (Table 4). The contribution of each individual mutation has yet to be assessed; however, these results confirmed that Inh-6 represents a novel genotype 2a-selective NS5A inhibitor.

**Table 4:** Resistance Studies.

	Inhibitor Name	Lifecycle Stage	Genotype Selectivity^a^	Fold Resistance^b^	HCV Proteins	Amino Acid Substitutions
Control Inhibitors	EI^e^	Entry	1a/1b	45^c^ (3)^d^	E2	V336G
	LY411575	Assembly	1a/1b/2a	None	-	-
Screen Hits	Inh-2	Entry	1a	>10 (3)	E1	L67F
				>10 (3)	E2	W333L
	Inh-4	Early	1a/1b/2a	None	-	-
	Inh-6	Replication	2a	30 (4)	NS5A	F28L, L31M, F169L
				100 (4)	NS5A	L31M, S38T, Q123R
	Inh-7	Replication	2a	>10 (4)	NS4B	G60S
	Inh-17	Late	1a/1b/2a	None	-	-

a Genotype coverage using gt 1a, 1b and 2a chimeric HCVcc.

b Virus cultured at 5× EC_50_ up to 10 weeks or until resistance observed.

c Mean fold resistance from 3 independent experiment.

d Time point (weeks) when resistance was observed.

e Baldick et al. 2009.

A single population exhibiting >10 fold resistance to Inh-7 was isolated and sequenced. A single G60S substitution in NS4B was identified, suggesting that Inh-7 was a novel genotype 2a-selective NS4B inhibitor. Resistance to Inh-4 or Inh-17 was not observed (Table 4). This finding coupled with the similar potencies observed against genotype 1a, 1b and 2a HCVcc chimeras suggests that these inhibitors may target cellular proteins. Taken together, these findings demonstrated that the gt 1a/2a-Rluc virus can be used for both screening and resistance studies and showed a clear correlation between targets identified through resistance and life cycle stage classification defined by the hit deconvolution strategy.

## Discussion

A number of clinical candidates and approved therapies for HCV include agents that target genome replication and have been advanced using the subgenomic HCV replicon (Reviewed in [42]). The recognized need for combination therapies for HCV creates a desire to identify inhibitors acting at other stages of virus infection, including those requiring the virus structural proteins. These stages include, at least, genome encapsidation, release of nascent virions from cells, and spread to and infection of new cells. While retroviral pseudo-particle assays can be used to identify inhibitors of virus entry [8], it is expected that the profile of some entry inhibitors could vary between pseudo-particles and authentic virus. In addition, other steps of entry including the eclipse or uncoating of the virion core, as well as later stages of infection, require a whole HCV virus assay for discovery.

Several HCV *in vitro* growth assays have been reported using the full-length genotype 2a or intragenotypic (2a) chimeric viruses [19], [20], [21], [22]. The Jc1 virus is highly amenable to assay development due to its ability to yield high titers (>1×10^6^ ffu/ml) and to rapidly spread through Huh-7.5 hepatocyte monolayers [18]. In our experience, however, significant effort can be required to evolve a lead inhibitor having activity against one HCV genotype to activity towards another. Since genotype 1 HCV is the most prevalent worldwide and is the least responsive to interferon-containing standard of care therapy, we were compelled to incorporate genotype 1 structural proteins into our HTS efforts. Since a full-length genotype 1 clones with robust growth properties does not yet exist [28], we focused our efforts on developing a growth assay using an intergenotypic chimera. The intergenotypic chimera used in this study produces a virus with the structural proteins (Core, E1 and E2) as well as p7 and NS2 of genotype 1a virus fused to NS3, NS4A, NS4B, NS5A and NS5B of a cell culture adapted JFH-1 virus. As a result, the entry process (E1 & E2), nucleocapsid uncoating (Core) and numerous aspects of virion assembly (e.g., Core, E1, E2, p7 and NS2) are directed by genotype 1 proteins. In contrast, replication of the viral RNA is directed exclusively by genotype 2 proteins (NS3-NS5B) although we cannot rule out unexpected and potentially trans-active roles for structural proteins, p7 or NS2 in the translation and replication of viral RNA in the context of complete genome replication.

Several key properties of the gt1a/2a-Rluc virus facilitated the development of an unbiased, high-throughput, whole-virus replication assay, many of which are detailed in a separate publication (Rose et al., manuscript in preparation). Firstly, the gt1a/2a-Rluc virus was modified molecularly and adapted in cell culture to yield sufficiently high titers (1–5×10^5^ ffu/ml) to accommodate the production of virus on a preparative scale. Secondly, the *Renilla* luciferase cassette located between NS5A and NS5B and bounded by authentic HCV protease cleavage sites yielded a genetically-stable virus, producing a robust signal whose amplification over time correlated directly with the spread of virus through the culture. Thirdly, this virus was selected for and adapted to spread in culture. Despite slower spreading kinetics relative to the Jc1 virus, following a 96 hour infection (MOI = 0.1), >90% of the total luciferase signal could be attributed to spreading virus. Importantly, this MOI was also sufficient to achieve a Z factor greater than 0.5 in a 384 well plate assay format, acceptable for HTS. Apoptosis of Huh-7.5 cells has been observed using highly replicating viruses (e.g., Jc1 virus), facilitating the development of screening formats using cell protection as a readout [19]. In contrast, replication of the gt 1a/2a-Rluc virus for 96 h in Huh-7.5 cells did not result in any measurable cytotoxicity (CellTiter-Glo assay) or reduce total cell numbers (Cellomics assay) suggesting that apoptosis did not have a significant impact on this assay (data not shown). Unbiased inhibitor detection was ensured as shown using control virus entry, genome replication and late-stage inhibitors that each blocked >90% of assay signal following 96 h in culture. Without a direct-acting anti-viral agent (DAA) targeting virus assembly, we used two approaches to recapitulate a late-stage inhibitor. In the first, the addition of the entry inhibitor EI which blocks both cell-free as well as cell-to-cell spread of virus [8], added following the first round of virus entry completely blocked subsequent infection and the spread of virus. We also used a signal peptide peptidase (SPP) inhibitor (LY411575) to perturb the maturation of Core which blocks the production of infectious virions [29], [30], [31], [32], [33], [34], [35], [36], [37], [38], [39], [40]. A large-scale screening campaign (>1 million compounds) was completed using *Renilla* Luciferase as readout and a comprehensive screening triage was implemented to rapidly identify potent and selective inhibitors. The development of a high-throughput HCV Core immunofluorescence Cellomics ArrayScan assay allowed us to confirm the HCV activity of leads independent of *Renilla* luciferase reporter activity. Although used as a secondary assay in this context, the HCVcc Cellomics assay will be a powerful tool to screen viruses without the need for a reporter which may prove particularly useful with less robust viruses that cannot accommodate a reporter gene.

Critical to any black box cell-based screening approach is the ability to rapidly segregate hits according to their target and genotype coverage. The combination of assays used in this study facilitated the identification of early, genome replication and late-stage inhibitors and allowed us to rank hits according to their activity against genotype 1 or 2 virus. While pan-genotype coverage is the goal of any HCV therapeutic, it was anticipated that most novel DAAs discovered in the screen would exhibit genotype selectivity unless targeting a highly conserved target in the virus or with a mode of action involving a cellular target. Consistent with this hypothesis, all of the early stage inhibitors exhibited selectivity for genotype 1 virus while the HCV selective genome replication inhibitors were selective for genotype 2. The late-stage inhibitors exhibited coverage of both genotype 1 and 2 HCVcc chimeras. However, it is unclear if these target the genotype 1 regions of the virus, the genotype 2 non-structural proteins shared by all of the chimeras, or cellular proteins.

The study of 17 chemically diverse compounds, representing structurally-unique clusters were chosen for profiling in this analysis; 5 early stage inhibitors, 8 genome replication inhibitors, and 4 late-stage inhibitors. Of the 5 early inhibitors, 3 exhibited similar potencies against both HCVcc and HCVpp pseudo-particles, each harboring H77 genotype 1a envelope glycoproteins (E1–E2). They had no activity against VSVpp pseudo-particles. Since the only HCV proteins contained in HCVpp are the envelope glycoproteins, these results suggest that the inhibitors target the viral proteins E1 and/or E2, or their functions are distinct from those involved in entry of VSVpp. Indeed, this was confirmed for Inh-2 as substitutions in either E1 or E2 conferred resistance to this compound. There are many possible explanations for the resistance to a single compound arising in either E1 or E2. The simplest is that mutation of a residue in one protein of the heterodimer can affect the conformation of the compound binding site in the other. Another straightforward possibility is that the compound binding interface is made up of both proteins. It also of interest that these mutations were selected independent of each other, in separate experiments, leading to questions as to whether or not they can co-exist on the same virus. Further studies are required to determine how this combination of mutations would affect viral fitness and resistance to Inh-2.

It is interesting to note that Inh-1 was active against only genotype 1a, while Inhs 2 and 3 were active against both genotype 1a and 1b but not 2a. Due to the genetic heterogeneity among HCV isolates belonging to different genotypes, broad spectrum antivirals with activity spanning across subtypes and genotypes are desired. Incomplete coverage by leads targeting both HCV structural [8], [9] as well as nonstructural [43] proteins has been reported. It is anticipated that medicinal chemistry efforts relating to the hits identified in this report might result in broad genotype coverage.

The other 2 early inhibitors had no activity against HCVpp suggesting that these could target an HCVcc-specific entry event or an aspect of the early phase of the life cycle not recapitulated in the HCVpp model. It is of interest that these inhibitors had no effect on transfected whole-genome vRNA or HCV replicons (data not shown) suggesting they could target a stage in the life cycle that occurs between envelope fusion and the initiation of vRNA replication such as nucleocapsid uncoating or the events directly preceding the initiation of vRNA translation/replication. Importantly, the identification of both entry and potentially post-entry/pre-replication inhibitors validated one of the goals of the infectious virus screening which was to probe novel targets previously inaccessible using other surrogate model systems.

Eight of the screen hits highlighted here exhibited a similar profile to a genome replication inhibitor (BMS-339; protease inhibitor) as they blocked HCVcc replication. Replication was blocked when initiated by infection or by transfection of vRNA. They also exhibited similar potency against a genotype 2a replicon demonstrating that these were bonafide genome replication inhibitors. Six of these hits are likely to be DAAs since they were inactive in HCV gt1 or BVDV replicon assays conducted in Huh7 cells. This was confirmed for Inhs 6 and 7 which selected for resistance substitutions exclusively within NS5A or NS4B, respectively. The remaining 2 inhibitors had similar potencies in HCVcc genotype 2a, 1a and BVDV replicon assays suggesting a common and likely cellular target shared within the replication machinery of these flaviruses. The final 4 inhibitors were confirmed to be late-stage inhibitors by their ability to block the production of infectious virus, but not replication. All of the inhibitors also blocked the replication of HCVcc chimeras with genotype 1a, 1b or 2a Core-NS2. As a result, it was not possible to discern if the activity was linked to the structural or non-structural proteins. The possibility exists that inhibitors targeting structural proteins could be active at both early and late stages of replication. Nonetheless, no inhibitors that were active at early or late stages demonstrated activity against both.

The selection and characterization of resistant viruses is a powerful tool to further understand the target, mechanism and spectrum of inhibitors. At the time this work was completed, we performed the selection of resistant viruses to a sampling of the HTS leads to demonstrate the utility and various results of the method. While inhibitors that target entry proteins and replication proteins can be readily selected, viruses resistant to inhibitors of host proteins were unable to be selected in these initial studies. Presumably selection conditions may be altered to enable selection for viruses that escape inhibition of host proteins, although it may be dependent on the particular host target [44], [45].

In summary, we leveraged a genotype 1a/2a intergenotypic HCVcc chimeric reporter virus, capable of relatively high titer replication, yield and spread to develop an infectious virus screening assay capable of testing large-scale compound libraries. We used an experimental triage process to identify inhibitors of various stages of the virus life cycle and confirmed the discovery of novel DAAs through resistance studies. Further optimization of these leads will be required on the path towards developing new DAAs to provide novel therapeutic combinations to inhibit HCV replication and prevent or manage the emergence of resistant virus.

## Materials and Methods

### Cells, culture conditions and reagents

293T cells (ATCC, Manassas, VA), Huh-H1 cells [8] and replicon cell lines were maintained in Dulbecco's Modified Eagle Medium (DMEM) (Invitrogen, Carlsbad, CA) supplemented with 10% fetal bovine serum (FBS) (HyClone, Logan, UT), 2 mM L-glutamine, 1 mM sodium pyruvate, 10% nonessential amino acids, 10 mM HEPES, 100 units/ml penicillin, and 100 units/ml streptomycin. Huh-7.5 cells (Apath, Brooklyn, NY) were maintained in DMEM containing 10% FBS, 10% nonessential amino acids, 100 units/ml penicillin, and 100 units/ml streptomycin. HCVcc infection assays were performed using DMEM containing 2% FBS, 10% nonessential amino acids, 100 units/ml penicillin, and 100 units/ml streptomycin (HCVcc infection media).

### Reporter virus construction

The full length JFH1 genome (accession number AB047639) was synthesized (DNA2.0 Inc., Menlo Park, CA) and assembled in plasmid pJ2 (DNA2.0 Inc., Menlo Park, CA) creating plasmid pJ2-JFH1. The J6CF-JFH1 chimeric virus, Jc1, was constructed as described [8] in the pJ2-JFH1 background creating plasmid pJ2-Jc1. A cell culture adapted Jc1 virus was selected having the substitutions E1 F100V, E2 V5G and E2 V31A and these changes were introduced back into pJ2-Jc1 creating plasmid pJ2-B7. To facilitate the cloning of reporter genes between NS5A and NS5B, pJ2-B7 was modified to contain a linker with three restrictions sites, BglII, XbaI and MluI, flanked by NS5A/NS5B cleavage sites between NS5A and NS5B. This was accomplished by PCR amplification of a small region of JFH1 flanking the NS5A/NS5B cleavage site in two fragments to introduce the restriction sites and the NS5A/NS5B cleavage site duplication. The 5′ fragment was PCR amplified with primers BR1332 (5′- CAT ATC AGA AGC CCT CCA GCA ACT-3′) and BR1385 (5′- GCT CTA GAA GAT CTC GAA TAG CTC ATA CTG CAG CAC ACG GTG GTA TCG TC-3′) and the 3′ fragment was amplified with primers BR1386 (5′- T CTA GAA CGC GTA GTG AAG AAG ATG ACA CAA CTG TAT GTT GTT CCA TGT CAT ACT CCT GGA CC-3′), BR1213 (5′- AAG CTC CCA TTA CCG CCT GAG-3′) using Pfu Ultra HF hot start polymerase (Agilent Technologies, La Jolla, CA). The 5′ fragment was digested with RsrII and XbaI and the 3′ fragment was digested with XbaI and HindIII. The digested 5′ and 3′ fragments were then ligated with a pJ2-B7 RsrII-HindIII fragment creating plasmid pJ2-B7-NS5A-BXM-NS5A. The human codon optimized *Renilla* luciferase (Rluc) gene from pGL4.70 (Promega, Madison WI) was PCR amplified with primers BR1387 (5′-TCT AGA AGA TCT ATG GCT TCC AAG GTG TAC GAC-3′) and BR1388 (5′- AAG CTT ACG CGT CTG CTC GTT CTT CAG CAC GCG CTC-3′) digested with BglII-MluI and ligated with pJ2-Jc1-B7-NS5A-BXM-NS5A digested with BglII-MluI creating plasmids pJ2-2a/2a-Rluc. This virus is referred to as the gt 2a/2a-Rluc virus.

To generate the 1a/2a-Rluc virus used in this study, the H-NS2/NS3-J virus (H77S Core-NS2 fused to JFH1 NS3-NS5B) was constructed as described [16] in pJ2-JFH1. Adaptive mutations, E1: Y361H and NS3: Q1251L, previously shown to enhance virus production from this molecular clone [16] were also included. Virus was further adapted by culture in Huh-7.5 cells until virus was recovered with enhanced spreading kinetics and the ability to yield titers exceeding 1×10^5^ focus forming unit (ffu)/ml. All of the substitutions observed from this virus population (E2: N576D, NS2: K927N, NS4B: I1901V, NS5A; V2106L and NS5A: S2357P) were introduced back into the parental virus and enhanced replication properties were confirmed. A 1.6 kb RsrII-HindIII fragment from pJ2-B7-NS5A-Rluc-NS5B having Rluc between NS5A and NS5B was cloned into a pJ2-1a/2a RsrII-HindIII fragment creating the plasmid pJ2-1a/2a-Rluc.

A HCV genotype 1b chimeric JFH1 virus consisting of core to NS2, up to the C3 junction [18] from a HCV genotype 1b isolate was constructed as described [8]. The genotype 1b sequence was obtained from a clinical isolate (Promeddx, Norton, MA) creating plasmid p432-4(1b)-JFH1. The resultant virus was adapted through passage in culture, resulting in the selection of substitutions E2: N532S and NS2: Y835C. These substitutions were introduced back to the parental virus plasmid, resulting in pJ2-1b/2a (GenBank accession number HM049503). A 1.6 kb RsrII-HindIII fragment from pJ2-B7-NS5A-Rluc-NS5B having Rluc between NS5A and NS5B was cloned into a pJ2-1b/2a RsrII-HindIII fragment creating the plasmid pJ2-1b/2a-Rluc. This virus is referred to as the gt 1b/2a-Rluc virus.

*In vitro* RNA was prepared from these cloned sequences using the MEGAscript kit (Ambion, Austin, TX) and transfected by electroporation into Huh-7.5 cells as described [46] Media containing virus was collected, clarified by low speed centrifugation, filtered through a Durapore 0.45 µm filter unit (Millipore, Billerica, MA) and stored at −80°C. HCVcc titers were determined by infection of Huh-7.5 cells with serial dilutions of virus, followed by indirect immunofluorescence for HCV core protein as described below, and expressed as focus forming units (ffu)/ml.

### HCVcc infection assays

Infections utilizing HCVcc expressing the *Renilla* luciferase protein were quantified by infecting Huh-7.5 cells (with or without inhibitors), incubating at 37°C for 2–4 days, and measuring luciferase activity using the EnduRen substrate (Promega) as described by the manufacturer. HCV Core immunostaining was used to directly visualize infectivity. HCVcc was added to Huh-7.5 cells (with or without inhibitors) in special-optics, collagen-coated 96-well plates (BD Biosciences) and incubated at 37°C for 2–4 days. Cells were then washed twice with PBS and fixed with 4% paraformaldehyde (Electron Microscopy Sciences, Hatfield, PA) in PBS for 30 min at room temperature. Following 2 washes in PBS, cells were permeabilized with 0.25% Triton X-100 (Pierce, Rockford, IL) in PBS for 10 min and blocked with 2% bovine serum albumen (Sigma) in PBS for 30 min. Samples were incubated for 2 hr with 3 µg/ml anti-HCV Core monoclonal antibody (ABR-Affinity Bioreagents, Golden, CO), washed 4 times with PBS, and incubated with a 1/500 dilution of Alexa Fluor 488-labeled donkey anti-mouse secondary antibody (Invitrogen) for 1 hr. Samples were washed three times with PBS and 0.5 µg/ml of Hoechst 33258 (Sigma) was added to the final wash to visualize nuclei. Infected cell foci were visualized using a Nikon Eclipse TE300 inverted epi-fluorescence microscope.

### High-throughput screening

Automated, large-scale culture of Huh-7.5 cells was performed using T-175 triple flasks (Thermo Scientific, Rochester, NY) on a SelectT automated cell culture system (Tap Biosystems, Greenville, DE). Large-scale virus stocks were prepared as above and maintained at −80°C until use. For compound library screening, infections were performed in 384-well plates by pre-mixing 400 ffu of gt 1a/2a-Rluc virus with 1×10^4^ Huh-7.5 cells and dispensing 50 µl on top of 150 nl of compound in DMSO (final 0.3% DMSO) followed by incubation at 37°C. *Renilla* luciferase activity, reflecting the degree of virus replication, was measured 96 h after infection using the EnduRen reagent (Promega). Luciferase activity was measured using an EnVision Multilabel Reader (Perkin Elmer), Test compounds were serially diluted 3-fold in DMSO to give a final concentration range in the assay of 60 µM to 3 nM. Maximum activity (100% of control) and background were derived from control wells containing DMSO alone or from uninfected wells, respectively. The individual signals in each of the compound test wells were then divided by the averaged control values (wells lacking inhibitor), after background subtraction, and multiplied by 100% to determine percent activity. The corresponding % inhibition values were then calculated by subtracting this value from 100. Dose-response assays were performed in triplicate and average EC_50_ values (reflecting the concentration at which 50% inhibition of virus replication was achieved) were calculated using XLfit for Excel (ID Business Solutions, Burlington, MA). Cytotoxicity studies were performed in parallel using CellTiter-Glo (Promega). For high-content screening, virus and cells were prepared as above and 50 ul was dispensed into black 384 well optics plates (Thermo Scientific). At 96 h post-infection, cells were fixed for 20 min by adding 17 ul of 16% paraformaldehyde (Electron Microscopy Sciences) to achieve 4% final fixative. Cells were washed and permeabilized twice for 10 min each with PBS containing 1% TX-100 and 0.05% Tween-20 (wash buffer) and blocked for 15 min with PBS with 2% BSA (blocking buffer). Cells were incubated for 1 hr with 3 µg/ml anti-HCV Core monoclonal antibody (ABR-Affinity Bioreagents) in 15 µl blocking buffer, washed 3 times with 100 µl of wash buffer and incubated with a 1/400 dilution of Alexa Fluor 488-labeled donkey anti-mouse secondary antibody (Invitrogen) in blocking buffer for 1 hr. Samples were washed three times with wash buffer and 0.5 µg/ml of Hoechst 33258 (Sigma) was added to the final wash to visualize nuclei. The total number of cells per well (Hoechst staining) and infected cells per well (HCV Core) was measured using a Cellomics ArrayScan HCS Reader (Thermo Scientific) and EC_50_ values calculated as described above. Corresponding Luciferase assays were performed on replica plates and reporter expression was normalized by calculating the relative light units per 100 infected cells.

### Pseudo-particle assays

Pseudo-particle infection assays were performed in 384-well plates by mixing HCVpp or VSVpp with 1×10^4^ Huh-H1 cells [8] per well in the presence or absence of test inhibitors, followed by incubation at 37°C. Huh-H1 cells over-express CD81 and infectivity by HCVpp is enhanced relative to Huh-7 cells [8]. Luciferase activity, reflecting the degree of entry of the pseudo-particles into host cells, was measured 48 h after infection using the Steady-Glo Reagent (Promega). All assays were performed in triplicate and average EC_50_ values were calculated using XLfit for Excel (ID Business Solutions, Burlington, MA).

### Viral RNA transfection and HCV replicon assays

*In vitro* transcribed gt 1a/2a-Rluc virus genomic viral RNA (vRNA) was electroporated into naïve Huh-7.5 cells and transfected cells were seeded into 384 well plates (10,000 cells/well) in the presence or absence of inhibitors. Luciferase activity, reflecting the degree of vRNA replication, was measured 24 h after transfection using the EnduRen Reagent (Promega). 10,000 cells were used in this format to increase the Luciferase signal per well as this assay was only 24 h in length compared to the viral infection assays which were either 48 or 96 h. Subgenomic replicon assays using genotypes 1a, 1b and 2a HCV and BVDV replicons have been described previously [47]. All assays were performed in triplicate and average EC_50_ values were calculated using XLfit for Excel (ID Business Solutions, Burlington, MA).

### HCVcc infectious virus release assay

Huh-7.5 cells were plated in 96-well plates at 1×10^4^ cells/well and infected 24 h later with gt 1a/2a-Rluc virus at an MOI>1 in the presence of inhibitors at 2× their respective EC_90_ values. Cells were incubated at 37°C for 48 h and supernatants were collected. Supernatants were clarified by centrifugation, diluted 20 fold and used to infect naïve Huh-7.5 cells in 96 well plates. Renilla luciferase expression was measured in both producer cells and target cells 48 h post-infection as described above.

### HCVcc resistance selection

The gt 1a/2a-Rluc virus was used to infect Huh-7.5 cells and inhibitor was added 12 h post-infection at 5× EC_50_. Infection was allowed to progress for 96 h and cells were split 1∶1 with naïve Huh-7.5 cells in the presence of inhibitor and cultured for another 96 h. Viral supernatants were passed onto naïve Huh-7.5 cells in the presence of inhibitor and HCVcc replication in the presence of inhibitor was monitored by determining the spread of virus infection, using immunofluorescence (HCV Core), at each passage. Generally, virus stocks were prepared when HCVcc was ≥10-fold resistant relative to wild-type parental virus. The HCV genome was amplified by RT-PCR (Invitrogen), cloned and amino acid changes that arose during inhibitor selection were identified by analysis of the DNA sequence compared to the parent and control passages in the absence of inhibitor.
